# Effect of liraglutide on cardiometabolic profile and on bioelectrical impedance analysis in patients with obesity and metabolic syndrome

**DOI:** 10.1038/s41598-023-40366-4

**Published:** 2023-08-11

**Authors:** Frederico Perboyre Carioca Freitas, Carlos Ewerton Maia Rodrigues

**Affiliations:** 1 Graduate Program in Medical Sciences, Universidade de Fortaleza (Unifor), Fortaleza, Brazil; 2 Department of Internal Medicine, Federal University of Ceará, Rua Gontran Giffoni 366, Apto 301, Torre I, Patriolino Ribeiro, Fortaleza, Ceará CEP 60810-220 Brazil

**Keywords:** Endocrinology, Medical research

## Abstract

Metabolic syndrome (MetS) and obesity represent a public health problem worldwide. Bioelectrical impedance analysis (BIA) is a practical and effective way of evaluating body composition, especially abdominal fat. Liraglutide, a GLP-1 analog, reduces body weight and improves cardiometabolic parameters. In this prospective non-randomized intervention study, we evaluated the effect of 6 months of treatment with liraglutide (n = 57) on the clinical, laboratory and BIA findings of adult sex-stratified patients diagnosed with obesity and MetS, compared to a control group receiving sibutramine (n = 46). The groups were statistically similar with regard to the age of females (*p* = 0.852) and males (*p* = 0.657). Almost all anthropometric and BIA variables were higher in the treatment group than in the comparative group (*p* < 0.05). Abdominal circumference (AC) decreased significantly more in the treatment group. In males, body weight and fat mass also decreased (*p* < 0.05). Liraglutide treatment was associated with a greater reduction in trunk fat mass (FMT) (*p* < 0.05). AC and FMT were strongly correlated (rho = 0.531, *p* < 0.001) in the treatment group. In the multiple regression analysis, liraglutide treatment remained independently associated with FMT. Treatment with liraglutide for 6 months promoted weight loss, improved cardiometabolic and inflammatory parameters and led to a significant reduction in FMT correlated with AC in obese MetS patients of both sexes.

## Introduction

Obesity is a complex and multifactorial chronic disorder frequently refractory to treatment and prediposing towards the development of cardiometabolic conditions, such as cardiovascular disease (CD), type-II diabetes mellitus (DM-II), systemic arterial hypertension (SAH), metabolic syndrome (MetS) and other comorbidities^1,2^.

MetS, a systemic proinflammatory condition, involves a set of complex metabolic changes, such as insulin resistance, central obesity, SAH, hypertriglyceridemia and reduced HDL cholesterol levels. Due to its close association with CD and DM-II, MetS is considered a major public health problem worldwide^3–6^.

Liraglutide is a glucagon-like peptide-1 (GLP-1) receptor agonist 97% similar to native GLP-1^7^ secreted by intestinal L-cells at the level of the distal jejunum, ileum and colon in response to the ingestion of carbohydrates, lipids and mixed food^8,9^. It reduces blood sugar levels, inhibits glucagon secretion, increases insulin secretion, suppresses the appetite and calorie intake, retards gastric emptying, and enhances sensitivity to insulin^10,11^.

Due to its direct implication for the metabolism, body composition should be determined before initiating treatment of obesity^12^. This may be done in the clinical setting by bioelectrical impedance analysis (BIA), a safe and simple procedure which provides timely results based on the measurement of electrical resistance in different body tissues^12^.

Few studies^13,14^ have used BIA in patients diagnosed with obesity and MetS, and to our knowledge no previous study has evaluated the effect of liraglutide on BIA parameters in obese patients with MetS. Ozhan et al.^13^ investigated the diagnostic performance of BIA in MetS and validated the best cut-off in a large adult cohort. The authors found that visceral fat measured with BIA is a useful and easily applicable method for identifying patients with MetS, using as cut-off values > 12% for men and > 9% for women. Likewise, Jeon et al.^14^ determined whether visceral fat area (VFA) measured by BIA was associated with MetS in subjects with and without obesity and demonstrated that BIA combined with body mass index (BMI) may be a useful target in interventions to improve MetS.

In this study, we evaluated the effect of 6 months of treatment with liraglutide on the clinical, laboratory and BIA parameters of adult patients diagnosed with obesity and MetS, stratified by sex, compared to a control group receiving sibutramine.

## Materials and methods

### Study approval

This prospective non-randomized intervention study of patient records was conducted at a private clinic in Fortaleza (Northeastern Brazil) from December 2021 to January 2023. The study complied with the tenets of the Declaration of Helsinki^15^, all activities were conducted in accordance with the approved protocols and guidelines, and all patients gave their informed written consent prior to inclusion in the study protocol. Submitted through an online national research database (Plataforma Brasil), the study protocol was approved by the Research Ethics Committee of University of Fortaleza (Unifor) and filed under entry #64954722.7.0000.5052.

### Patients and inclusion and exclusion criteria

The trial enrolled 103 patients of both sexes aged ≥ 21 years, with a BMI (BMI = body mass divided by the square of the body height) of 30 kg/m^2^ or higher and a diagnosis of MetS based on the “Harmonizing the Metabolic Syndrome” criteria (IDF/NHLBI/AHA/WHO/IAS/IASO) adjusted for South Americans^16^ and stratified by sex. Over a period of 6 months, 57 eligible patients received liraglutide at 3 mg/day s.c. (treatment group) and 46 eligible patients received sibutramine at 15 mg/day p.o. (control group). Patients were selected by convenience sampling and participants in both groups were aware of the medication received. All patients were submitted to physical examination, BIA and lab testing at baseline and at 6 months, stratified by sex.

Liraglutide is marketed under the trade name Saxenda by Novo Nordisk A/S (Bagsværd, Denmark) and Novo Nordisk Pharmaceutical Industries LP (Clayton, USA). Neither company was involved in this study, or supported it in any manner, or had access to the study data. The compound, a GLP-1 receptor agonist, reduces the appetite and, consequently, reduces food ingestion, promoting weight loss. The drug can cause nausea, vomiting, diarrhea, constipation, loss of appetite, dyspeptic symptoms, sensation of weakness, injection site reactions (hematoma, irritation, rash) and dizziness, among other effects^17^.

Sibutramine hydrochloride monohydrate is an anti-obesity drug which acts primarily through its active metabolites monodesmethyl (M1) and didesmethyl (M2) by effectively blocking the recapture of serotonin (5-hydroxytryptamine), norepinephrine and dopamine. The compound inhibits the appetite by promoting a sensation of satiety and diminishes weight loss-induced decline in energy expenditure^18^. The adverse effects include constipation, dry mouth and insomnia (up to 10% of cases), and palpitations, tachycardia, headache, increased blood pressure and sweating (less than 10% of cases). The brand Biomag was used in this study. The manufacturer (Achè Laboratórios Farmacêuticos S.A.) did not support this study in any manner and had no access to the study data.

The general exclusion criteria were age < 21 years, BMI < 30 kg/m^2^, MetS diagnosed by criteria other than the “Harmonizing the Metabolic Syndrome” criteria adjusted for South Americans^16^, patients with hypothyroidism, depression, use of antidepressants, obstructive sleep apnea, pregnancy and breastfeeding. Moreover, in the treatment group we also excluded patients with contraindications to liraglutide (history of hypersensitivity to the drug, age > 75 years, pancreatitis, multiple endocrine neoplasia, family history of medullary carcinoma of the thyroid). In the comparative group we excluded patients with contraindications to sibutramine (hypersensitivity to sibutramine, > 65 years of age, history of acute myocardial infarction, congestive heart failure, arrhythmia, peripheral arterial occlusive disease, treatment of psychiatric disorders, poorly controlled hypertension and/or previous cerebrovascular disease).

### Study protocol

All patients were submitted to clinical and anthropometric evaluations, including abdominal circumference (AC), arterial pressure and lab tests, at baseline and after 6 months of protocol. Patients in the treatment group were instructed in the proper daily subcutaneous administration of liraglutide (preferably in the morning, in the abdomen or the upper inner arm) at an initial dose of 0.6 mg/day. The dose was raised by 0.6 mg at weekly intervals until reaching 3 mg/day (0.6 → 1.2 → 1.8 → 2.4 → 3 mg/day). The comparative group received sibutramine at 15 mg/day p.o. in the morning. Patients were monitored for pharmacological tolerance, including adverse effects like nausea, vomiting, diarrhea, constipation, loss of appetite, dyspepsia, sensation of weakness, injection site reactions (hematoma, irritation, rash), dizziness or palpitations, tachycardia, headache, and increased blood pressure. All patients were instructed to reduce their calorie ingestion and to perform 150–300 min of moderately intensive or 75–150 min of vigorous physical activity per week, or an equivalent combination thereof^19^.

### Clinical, anthropometric and laboratory evaluations

During the clinical examination, a standardized questionnaire was administered to collect personal information on current health, food habits, physical activity, current and previous treatments, comorbidities, and family history of obesity, diabetes and SAH.

AC was measured with a tape positioned horizontally halfway between the iliac crest and the last rib. Height was measured using a digital stadiometer (HM-210 D, Ottoboni^®^).

Arterial pressure was measured with a previously calibrated sphygmomanometer, using a cuff compatible with the patient’s arm circumference (cuff size 12 × 23 for 25–34 cm; cuff size 16 × 32 for 35–45 cm). After resting for at least 5 min in a quiet room, arterial pressure was measured twice at a minimum interval of 2 min, as proposed by the 2018 ESH/ESC guidelines for the management of SAH^20^.

Body weight, segmental fat mass and segmental lean mass were quantified for all body segments (arms, legs, trunk) using an InBody 270 tetrapolar bioimpedance device^21^ manufactured in South Korea and licensed in Brazil by Anvisa under #80051870004. To do so, the patient was positioned on a scale (InBody 270), with electrodes attached to the hands and feet. The results were reported as percentage of body fat (BF%), lean mass, weight, body water and BMI. The test is painless and takes less than 5 min^21^.

The bioimpedance device features 8 contact points capable of collecting 10 measurements from each body segment (right arm, left arm, right leg, left leg, trunk) using 2 different frequencies (20 kHz and 100 kHz) and a current of 250 µA (Table 1)^22^. For the best results, patients were recommended to abstain from food and drink 2 h before the evaluation, void the bladder immediately before, not to practice physical activity or use the sauna on the day of the evaluation, and not to be menstruating. Evaluations were conducted at room temperature (20–25 °C).

**Table 1:** Bioimpedance parameters registered in the study.

Parameter	Abbreviation
Total weight	TWT
Fat mass in the right arm	FMA-R
Fat mass in the left arm	FMA-L
Fat mass in the trunk	FMT
Fat mass in the right leg	FML-R
Fat mass in the left leg	FML-L
Lean mass in the right arm	LMA-R
Lean mass in the left arm	LMA-L
Lean mass in the trunk	LMT
Lean mass in the right leg	LML-R
Lean mass in the left leg	LML-L
Body-mass index	BMI
Percentage of body fat	FM%
Waist-to-hip ratio	WHR

Blood was collected after 12 h of fasting and 72 h of abstention from alcohol and heavy exercise. The lab parameters included fasting glycemia, insulin, glycated hemoglobin, HOMA-IR, total cholesterol, HDL, LDL, triglycerides, uric acid, C-reactive protein (CRP) and erythrocyte sedimentation rate (ESR).

ESR (mm/hr) was measured in whole blood using the automated Westergreen method. CRP (mg/dL) was measured in serum on nephelometry (Dade-Behring^®^ BNII). Serum levels of glucose (mg/dL) were determined with the glucose-oxidase enzyme method, while serum levels of urea (mg/dL) were estimated with the UV-kinetic method. Using the kinetic method without deproteinization, we quantified serum creatinine (mg/dL), while enzymatic colorimetry was employed to determine the level of triglycerides (mg/dL), total cholesterol (mg/dL) and uric acid (mg/dL). To obtain the lipid profile (mg/dL), we submitted serum samples to calorimetry (Wiener^®^CMD 800i; Konelab^®^60i). Serum was also used for the estimation of high-density lipoprotein (HDL) (mg/dL) and low-density lipoproteine (LDL) (mg/dL) on calorimetry (calculated with the Fredwald formula CT = HDL + LDL + TG/5 whenever triglycerides were < 300 mg/dL). Finally, the insulin concentration in whole blood (µU/mL) was estimated on immunofluorometry and insulin resistence was defined by the HOMA-IR index of the top quartile of a non-diabetic population^16^.

### Diagnosis of metabolic syndrome

MetS was classified according to the “Harmonizing the Metabolic Syndrome” statement (IDF/NHLBI/AHA/WHO/IAS/IASO)^16^, which requires the presence of 3 of the 5 criteria below:

- Increase in AC using values adjusted for South Americans (≥ 90 for men; ≥ 80 for women)
- TG ≥ 150 mg/dL, or receiving treatment
- HDL ≤ 40 mg/dL for men and ≤ 50 mg/dL for women, or receiving treatment
- Arterial pressure ≥ 130/ ≥ 85 mmHg, or use of antihypertensive medication
- Fasting glycemia ≥ 100 mg/dL, or diagnosis of DM.

### Statistical analysis

Categorical variables were expressed as absolute values and relative frequency (%). The chi-squared test was used to identify associations between categorical variables. The normality of distribution of the continuous variables was verified with the Kolmogorov–Smirnov test. Asymmetry was evaluated based on histograms and Q-Q graphs. Normal data were expressed as means ± standard deviation, while non-normal data were expressed as medians and interquartile range.

Pairwise comparisons of continuous variables of independent groups were made with Student’s *t* test (normal distribution) or the Mann–Whitney test (non-normal distribution). Pairwise comparisons of dependent groups were made with the paired *t* test (normal distribution) or the Wilcoxon test (non-normal distribution). Finally, quantitative variables were submitted to Spearman’s non-parametric correlation analysis (*rho* coefficient).

Furthermore, multiple linear regressions were conducted to verify the existence of independent associations between the study variables and reduction in FMT at 6 months (dependent variable), the most significant parameter in the univariate analysis. All parameters significant at the level of 10% (*p* < 0.10) in the univariate analysis were tested by multivariate analysis. Additionally, sex and age were included as independent variables. Collinearity between quantitative variables was assessed. All the variables selected for the multivariate model were included manually, and a backward stepwise method was used to identify the model which best explained the observed changes in the dependent variable.

All statistical analyses were performed with the software SPSS for Macintosh v. 23 (Armonk, NY: IBM Corp.). The level of statistical significance was set at 5% (*p* < 0.05).

## Results

Our sample of MetS patients (n = 103) was segregated into a treatment group (n = 57, liraglutide 3 mg/day) and a comparative group (n = 46, sibutramine 15 mg/day). The groups were stratified according to sex: females accounted for 26 patients in the comparative group and 24 in the treament group, while males accounted for 20 patients in the comparative group and 33 in the treament group. The groups did not differ statistically with regard to female age (*p* = 0.852) and male age (*p* = 0.657). Almost all anthropometric variables were higher in the treatment group than in the comparative group (*p* < 0.05) regardless of the sex, with the exception of muscle mass (both sexes) and waist-to-hip ratio (males) (Table 2).

**Table 2:** Baseline findings for the treatment group (liraglutide) and the control group (sibutramine).

	Female		*p*	Male		*p*
	Control (n = 26)	Liraglutide (n = 24)		Control (n = 20)	Liraglutide (n = 33)	
Clinical findings						
Age (years)	46 ± 10	46 ± 9	0.852	43 ± 11	42 ± 11	0.657
SAP (mmhg)	121 ± 5.7	129.8 ± 14.3	0.008	121.5 ± 7.1	129.8 ± 9.9	0.002
DAP (mmhg)	80.6 ± 4.3	81.3 ± 8	0.716	79 ± 4.5	84.8 ± 5.8	< 0.001
Anthropometric findings						
Body weight (kg)	81 ± 8	92 ± 12	0.001	103 ± 20	117 ± 18	0.016
Muscle mass (kg)	24.2 ± 4.5	25.3 ± 3.3	0.35	37.4 ± 5.6	39.3 ± 4.2	0.16
Fat mass (kg)	37.2 ± 6.8	45.7 ± 8.9	< 0.001	37.3 ± 13.8	47.8 ± 14.5	0.012
BMI (kg/m^2^)	28.2 ± 2.9	36.3 ± 3.9	< 0.001	30.6 ± 4	38.3 ± 5.8	0.008
AC (cm)	99.7 ± 6.9	109.9 ± 10	< 0.001	114.5 ± 15.7	128.5 ± 14.5	0.002
WHR	1 ± 0	1 ± 0.1	0.008	1.1 ± 0.1	1.1 ± 0.1	0.23
Bioimpedance						
FM%	45.9 ± 5.7	49.7 ± 4.9	0.016	35.3 ± 6.3	40.1 ± 7.1	0.016
LMA-R (kg)	2.1 ± 0.5	2.5 ± 0.5	0.014	3.4 ± 1	3.8 ± 0.9	0.152
LMA-L (kg)	2.1 ± 0.5	2.5 ± 0.5	0.026	3.5 ± 0.9	3.9 ± 0.8	0.17
LMT (kg)	17 ± 4.4	19.6 ± 4.2	0.036	24.4 ± 8.2	26.9 ± 6.8	0.228
LML-R (kg)	5.9 ± 0.9	6.6 ± 1.2	0.025	8.6 ± 2.1	9.2 ± 1.9	0.279
LML-L (kg)	6 ± 1	6.6 ± 1.2	0.037	8.6 ± 2.2	9.2 ± 1.9	0.281
FMA-R (kg)	3.1 ± 1.2	4 ± 1.2	0.005	2.9 ± 1.6	4.4 ± 2	0.004
FMA-L (kg)	2.9 ± 1	4.1 ± 1.2	0.001	2.9 ± 1.6	4.4 ± 2	0.003
FMT (kg)	19.3 ± 4.1	24.1 ± 4.9	< 0.001	20.6 ± 6.8	25.8 ± 5.9	0.005
FML-R (kg)	5.7 ± 1.1	6.9 ± 1.5	0.003	5.5 ± 2.1	7 ± 2.5	0.028
FML-L (kg)	5.8 ± 1.2	7 ± 1.6	0.003	5.5 ± 2.1	7 ± 2.5	0.03
Laboratory findings						
Fasting glycemia (mg/dL)	96.7 ± 10.2	100.2 ± 15.7	0.344	95.5 ± 9.4	98.2 ± 16.4	0.509
Insulin (µU/mL)	12.2 (9.4–20.4)	20.7 (9.4–26.5)	0.171	17.5 (10.5–22)	20.2 (13.9–27)	0.132
HOMA-IR	2.71 (2.01–4.9)	5.47 (2.25–6.7)	0.116	4.24 (2.42–5.35)	4.89 (3.4–6.7)	0.125
Total cholesterol (mg/dL)	197.3 ± 40.5	205.4 ± 46.6	0.514	190.7 ± 63.5	189.2 ± 40.1	0.918
LDL (mg/dL)	127.1 ± 38	127.8 ± 42	0.953	132.4 ± 44.2	121.7 ± 39.6	0.369
HDL (mg/dL)	46.3 ± 11.1	57.2 ± 21.8	0.034	44.1 ± 21.5	44.2 ± 15.2	0.982
Triglycerides (mg/dL)	178.8 ± 56	207.3 ± 80.5	0.156	220.7 ± 80.7	202.9 ± 71.1	0.406
TGO (U/L)	27.5 (23–32)	22.5 (16.5–27.5)	0.021	26 (22–30.5)	34 (28–44)	0.016
TGP (U/L)	25 (22–31)	28 (18–41.5)	0.800	30 (24.5–41)	46 (28–71)	0.022
Urea (mg/dL)	31.7 ± 9.1	27.7 ± 8	0.101	36.2 ± 5.1	33.5 ± 8.4	0.219
Creatinine (mg/dL)	0.77 ± 0.17	0.77 ± 0.14	0.970	0.94 ± 0.15	0.92 ± 0.18	0.603
Vitamin D (ng/mL)	29.8 ± 10.8	26.9 ± 9.9	0.333	32.5 ± 14.6	25.6 ± 7.6	0.061
ESR (mm/h)	11.5 (7–17)	12 (9–18)	0.250	16 (11.5–22)	17 (9–21.5)	0.748
CRP (mg/dL)	0.29 (0.08–0.72)	0.77 (0.41–2.01)	0.005	0.28 (0.14–0.94)	0.63 (0.3–1.11)	0.128
TSH (U/L)	2.37 ± 1.1	1.82 ± 0.79	0.045	1.8 ± 0.67	1.91 ± 0.77	0.598
Glycated hemoglobin (%)	5.4 ± 0.5	5.8 ± 0.9	0.095	5.5 ± 0.5	5.8 ± 1.3	0.281
Uric acid (mg/dL)	4.7 ± 1	4.7 ± 1.3	0.808	6 ± 1.3	6.7 ± 1.7	0.122

Continuous variables expressed as mean ± standard deviation or median and interquartile range (in parenthesis). Categorical variables were expressed as absolute values and percentages (in parenthesis). Continuous variables were compared with Student’s *t* test or the Mann–Whitney test. Categorical variables were analyzed with the chi-square test.

*SAP* Systolic arterial pressure, *DAP* Diastolic arterial pressure, *BMI* Body-mass index, *AC* Abdominal circumference, *WHR* Waist-to-hip ratio, *FM%* Percentage of body fat, *LMA-R* Lean mass in the right arm, *LMA-L* Lean mass in the left arm, *LMT* Lean mass in the trunk, *LML-R* Lean mass in the right leg, *LML-L* Lean mass in the left leg, *FMA-R* Fat mass in the right arm, *FMA-L* Fat mass in the left arm, *FMT* Fat mass in the trunk, *FML-R* Fat mass in the right leg, *FML-L* Fat mass in the left leg, *HOMA-IR* Homeostatic Model Assessment for Insulin Resistance, *LDL* Low-density lipoprotein, *HDL* High-density lipoprotein, *TGO* Aspartate aminotransferase, *TGP* Alanine aminotransferase, *ESR* Erythrocyte sedimentation rate, *CRP* C-reactive protein, *TSH* Thyroid-stimulating hormone.

Likewise, at baseline all BIA variables in females were significantly higher in the treatment group than in the comparative group (*p* < 0.05). As for males, many of the BIA variables were statistically similar at baseline, such as lean mass in the right arm (LMA-R) (*p* = 0.152), lean mass in the left arm (LMA-L) (*p* = 0.170), lean mass trunk (LMT) (*p* = 0.228), lean mass in the right leg (LML-R) (*p* = 0.279) and lean mass in the left leg (LML-L) (*p* = 0.281).

Among laboratory variables, females treated with liraglutide had higher levels of HDL (*p* = 0.034) and CRP (*p* = 0.005) than females treated with sibutramine (Table 2).

Treatment with liraglutide at 3 mg/day for 6 months significantly improved all clinical and anthropometric variables (*p* < 0.05) and most BIA variables in both sexes. Females treated with liraglutide had a lower body fat percentage (FM%), LMA-R, LMA-L, fat mass in the right arm (FMA-R), fat mass in the left arm (FMA-L), fat mass in the trunk (FMT), fat mass in the right leg (FML-R) and fat mass in the left leg (FML-L) (*p* < 0.05). In males, the decreased variables included FM%, LMA-R, LMA-L, LML-R, LML-L, FMA-R, FMA-L, FMT, FML-R and FML-L (*p* < 0.05) (Table 3).

**Table 3:** Clinical, anthropometric and bioimpedance findings at baseline and after 6 months of treatment with liraglutide.

	Female		*p*	Male		*p*
	Liraglutide (n = 24)			Liraglutide (n = 33)		
	Baseline	6 months		Baseline	6 months	
Clinical parameters						
SAP (mmhg)	129.8 ± 14.3	111 ± 10.4	< 0.001	129.8 ± 9.9	111.8 ± 8.1	< 0.001
DAP (mmhg)	81.3 ± 8	71.3 ± 9	< 0.001	84.8 ± 5.8	72.4 ± 7.9	< 0.001
Anthropometric parameters						
Body weight (kg)	92 ± 12	80.6 ± 10.4	< 0.001	117 ± 18	103.4 ± 16.7	< 0.001
Muscle mass (kg)	25.3 ± 3.3	24.2 ± 2.7	0.007	39.3 ± 4.2	37.9 ± 4.2	< 0.001
Fat mass (kg)	45.7 ± 8.9	35.8 ± 8.9	< 0.001	47.8 ± 14.5	34.6 ± 14.1	< 0.001
BMI (kg/m^2^)	36.3 ± 3.9	31.4 ± 3.7	< 0.001	38.3 ± 5.8	33.8 ± 5.5	< 0.001
AC (cm)	109.9 ± 10	98.3 ± 8.2	< 0.001	128.5 ± 14.5	114.4 ± 12.9	< 0.001
WHR	1 ± 0.1	1 ± 0	< 0.001	1.1 ± 0.1	1 ± 0.1	< 0.001
Bioimpedance						
FM%	49.7 ± 4.9	43.9 ± 6.9	< 0.001	40.1 ± 7.1	33.9 ± 8.6	< 0.001
LMA-R (kg)	2.5 ± 0.5	2.2 ± 0.6	0.004	3.8 ± 0.9	3.5 ± 0.9	0.001
LMA-L (kg)	2.5 ± 0.5	2.2 ± 0.6	0.009	3.9 ± 0.8	3.6 ± 0.8	< 0.001
LMT (kg)	19.6 ± 4.2	18.4 ± 4.3	0.053	26.9 ± 6.8	26.2 ± 6.7	0.053
LML-R (kg)	6.6 ± 1.2	6.3 ± 1.3	0.063	9.2 ± 1.9	8.6 ± 1.8	< 0.001
LML-L (kg)	6.6 ± 1.2	6.3 ± 1.3	0.058	9.2 ± 1.9	8.7 ± 1.9	< 0.001
FMA-R (kg)	4 ± 1.2	3 ± 1.1	< 0.001	4.4 ± 2	3.4 ± 1.8	< 0.001
FMA-L (kg)	4.1 ± 1.2	3.1 ± 1.1	< 0.001	4.4 ± 2	3.4 ± 1.9	< 0.001
FMT (kg)	24.1 ± 4.9	17.1 ± 4.5	< 0.001	25.8 ± 5.9	18.4 ± 5.4	< 0.001
FML-R (kg)	6.9 ± 1.5	5.5 ± 1.1	< 0.001	7 ± 2.5	5.9 ± 2.4	< 0.001
FML-L (kg)	7 ± 1.6	5.6 ± 1.1	< 0.001	7 ± 2.5	6 ± 2.4	< 0.001

Continuous variables were compared with the pared *t* test and expressed as mean ± standard deviation.

*SAP* Systolic arterial pressure, *DAP* Diastolic arterial pressure, *BMI* Body-mass index, *AC* Abdominal circumference, *WHR* Waist-to-hip ratio, *FM%* Percentage of body fat, *LMA-R* Lean mass in the right arm, *LMA-L* Lean mass in the left arm, *LMT* Lean mass in the trunk, *LML-R* Lean mass in the right leg, *LML-L* Lean mass in the left leg, *FMA-R* Fat mass in the right arm, *FMA-L* Fat mass in the left arm, *FMT* Fat mass in the trunk, *FML-R* Fat mass in the right leg, *FML-L* Fat mass in the left leg.

Likewise, most laboratory parameters improved in the treatment group, regardless of the sex, as did the inflammatory parameters ESR (*p* < 0.05) and CRP (*p* = 0.05) (Table 4).

**Table 4:** Laboratory findings at baseline and after 6 months of treatment with liraglutide.

	Female		*p*	Male		*p*
	Liraglutide (n = 24)			Liraglutide (n = 33)		
	Baseline	6 months		Baseline	6 months	
Laboratory parameters						
Fasting glycemia (mg/dL)	100.2 ± 15.7	85.3 ± 8.7	< 0.001	98.2 ± 16.4	88.7 ± 9.2	< 0.001
Insulin (µU/mL)	20.7 (9.4–26.5)	9.4 (6.4–14.3)	< 0.001	20.2 (13.9–27)	12 (9.2–16.7)	< 0.001
HOMA-IR	5.47 (2.25–6.7)	2.15 (1.39–2.92)	< 0.001	4.89 (3.4–6.7)	2.4 (1.91–3.8)	< 0.001
Total cholesterol (mg/dL)	205.4 ± 46.6	177.4 ± 31.8	0.008	189.2 ± 40.1	169.1 ± 34.3	0.002
LDL (mg/dL)	127.8 ± 42	97.7 ± 33.4	0.015	121.7 ± 39.6	97 ± 29	< 0.001
HDL (mg/dL)	57.2 ± 21.8	59 ± 21.7	0.785	44.2 ± 15.2	51.2 ± 15	0.059
Triglycerides (mg/dL)	207.3 ± 80.5	108.5 ± 49.2	< 0.001	202.9 ± 71.1	109.8 ± 40.8	< 0.001
TGO (U/L)	22.5 (16.5–27.5)	21 (16–26)	0.253	34 (28–44)	24 (22–30)	< 0.001
TGP (U/L)	28 (18–41.5)	21 (18–30)	0.027	46 (28–71)	33 (26–47)	< 0.001
Urea (mg/dL)	27.7 ± 8	30.1 ± 5.6	0.171	33.5 ± 8.4	32.7 ± 9.9	0.505
Creatinine (mg/dL)	0.77 ± 0.14	0.8 ± 0.18	0.303	0.92 ± 0.18	0.95 ± 0.16	0.413
Vitamin D (ng/mL)	26.9 ± 9.9	33.3 ± 10.7	0.039	25.6 ± 7.6	30.6 ± 5.7	0.006
ESR (mm/h)	12 (9–18)	8 (7–11)	0.003	17 (9–21.5)	9 (6–11)	< 0.001
CRP (mg/dL)	0.77 (0.41–2.01)	0.48 (0.21–0.69)	0.004	0.63 (0.3–1.11)	0.45 (0.16–0.7)	0.001
TSH (U/L)	1.82 ± 0.79	1.86 ± 0.51	0.729	1.91 ± 0.77	1.67 ± 0.71	0.157
Glycated hemoglobin (%)	5.8 ± 0.9	5.4 ± 0.4	0.001	5.8 ± 1.3	5.2 ± 0.5	0.015
Uric acid (mg/dL)	4.7 ± 1.3	4.4 ± 0.9	0.176	6.7 ± 1.7	5.5 ± 1.4	< 0.001

Continuous variables were compared with the paired *t* test or the Mann–Whitney test and expressed as mean ± standard deviation or median and interquartile range (in parenthesis).

*HOMA-IR* Homeostatic Model Assessment for Insulin Resistance, *LDL* Low-density lipoprotein, *HDL* High-density lipoprotein, *TGO* Aspartate aminotransferase, *TGP* Alanine aminotransferase, *ESR* Erythrocyte sedimentation rate, *CRP* C-reactive protein, *TSH* Thyroid-stimulating hormone.

Subsequently, the two groups were compared with regard to changes in clinical, anthropometric, laboratory and BIA parameters, stratified by sex. Weight loss (5% and 10%) was similar in both sexes, but in women AC decreased significantly more in the treatment group than in the comparative group (− 11 [− 14.5; − 8.0] vs − 5 [− 7; − 4] cm, *p* < 0.001), while in men a decrease was observed in body weight (− 12.6 [− 17.5; − 10] vs − 9.8 [− 14.3; − 6.7] cm, *p* = 0.037), fat mass (− 10.9 [− 14.8; − 8.5] vs − 8.5 [− 11.1; − 5.45] cm, *p* = 0.010) and AC (− 14 [− 16; − 11] vs − 7 [− 11.2; − 4] cm, *p* < 0.001) (Table 5).

**Table 5:** Comparison of bioimpedance and anthropometric variables in the treatment group (liraglutide) and the control group (sibutramine).

Variation (post–pre-treatment)	Female		*p*	Male		*p*
	Control (n = 26)	Liraglutide (n = 24)		Control (n = 20)	Liraglutide (n = 33)	
Loss of 5% of baseline weight			0.34			0.549
No	1 (3.8)	3 (12.5)		2 (10)	1 (3)	
Yes	25 (96.2)	21 (87.5)		18 (90)	32 (97)	
Loss of 10% of baseline weight			0.902			0.152
No	8 (30.8)	7 (29.2)		10 (50)	10 (30.3)	
Yes	18 (69.2)	17 (70.8)		10 (50)	23 (69.7)	
Clinical parameters						
SAP (mmhg)	− 10 (− 10; 0)	− 20 (− 30; − 10)	0.003	0 (− 5; 0)	− 20 (− 30; − 10)	< 0.001
DAP (mmhg)	− 5 (− 10; 0)	− 10 (− 20; − 5)	0.157	0 (− 2.5; 0)	− 10 (− 20; − 10)	< 0.001
Anthropometric parameters						
Body weight (kg)	− 9.25 (− 11.5; − 7.2)	− 11.1 (− 15.2; − 6.5)	0.203	− 9.8 (− 14.35; − 6.75)	− 12.6 (− 17.5; − 10)	0.037
Muscle mass (kg)	− 1.15 (− 1.8; 0)	− 0.7 (− 2.1; 0.1)	0.808	− 1 (− 1.8; − 0.3)	− 1.2 (− 2.8; − 0.6)	0.287
Fat mass (kg)	− 6.5 (− 9.8; − 5.1)	− 8.55 (− 14; − 6.8)	0.052	− 8.5 (− 11.1; − 5.45)	− 10.9 (− 14.8; − 8.5)	0.010
BMI (kg/m^2^)	− 3.6 (− 4.6; − 3.1)	− 4.7 (− 6.2; − 3.45)	0.091	− 3.25 (− 4.75; − 2.4)	− 4.4 (− 5.6; − 3.5)	0.064
AC (cm)	− 5 (− 7; − 4)	− 11 (− 14.5; − 8)	< 0.001	− 7 (− 11.25; − 4)	− 14 (− 16; − 11)	< 0.001
WHR (cm)	− 0.05 (− 0.07; − 0.04)	− 0.08 (− 0.1; − 0.04)	0.136	− 0.06 (− 0.09; − 0.05)	− 0.08 (− 0.1; − 0.06)	0.302
Bioimpedance						
FM%	− 4.6 (− 7.2; − 3)	− 5.45 (− 9.05; − 2.6)	0.816	− 5.15 (− 7.5; − 2.95)	− 5.7 (− 7.9; − 4.4)	0.326
LMA R (kg)	− 0.2 (− 0.3; 0)	− 0.25 (− 0.4; − 0.1)	0.469	− 0.2 (− 0.4; − 0.05)	− 0.2 (− 0.4; 0)	0.832
LMA-L (kg)	− 0.2 (− 0.3; − 0.1)	− 0.2 (− 0.4; − 0.1)	0.791	− 0.25 (− 0.35; − 0.05)	− 0.2 (− 0.5; 0)	0.971
LMT (kg)	− 0.75 (− 1.5; 0)	− 1 (− 2.3; − 0.15)	0.484	− 0.7 (− 1.2; − 0.05)	− 1 (− 1.8; 0)	0.312
LML-R (kg)	− 0.2 (− 0.4; 0)	− 0.2 (− 0.6; 0)	0.458	− 0.3 (− 0.5; 0)	− 0.5 (− 0.8; − 0.1)	0.188
LML-L (kg)	− 0.25 (− 0.5; 0)	− 0.2 (− 0.6; 0)	0.640	− 0.4 (− 0.5; 0)	− 0.5 (− 0.8; − 0.2)	0.109
FMA-R (kg)	− 0.8 (− 1.2; − 0.5)	− 0.85 (− 1.6; − 0.5)	0.526	− 0.7 (− 1; − 0.4)	− 1.1 (− 1.5; − 0.7)	0.024
FMA-L (kg)	− 0.7 (− 1.1; − 0.4)	− 0.85 (− 1.55; − 0.4)	0.471	− 0.7 (− 1.05; − 0.4)	− 1 (− 1.4; − 0.7)	0.049
FMT (kg)	− 3.15 (− 4.9; − 2.5)	− 5.85 (− 9.7; − 4.2)	0.001	− 3.85 (− 6.15; − 3.1)	− 7.8 (− 9.4; − 6.5)	< 0.001
FML-R (kg)	− 1.05 (− 1.4; − 0.7)	− 1.3 (− 1.6; − 0.85)	0.307	− 1.35 (− 1.65; − 0.8)	− 1.1 (− 1.5; − 0.8)	0.672
FML-L (kg)	− 1 (− 1.4; − 0.7)	− 1.2 (− 1.5; − 0.85)	0.335	− 1.35 (− 1.7; − 0.8)	− 1.1 (− 1.6; − 0.8)	0.620

Continuous variables were compared with the Mann–Whitney test and expressed as median and interquartile range.

*SAP* Systolic arterial pressure, *DAP* Diastolic arterial pressure, *BMI* Body-mass index, *AC* Abdominal circumference, *WHR* Waist-to-hip ratio, *FM%* Percentage of body fat, *LMA-R* Lean mass in the right arm, *LMA-L* Lean mass in the left arm, *LMT* Lean mass in the trunk, *LML-R* Lean mass in the right leg, *LML-L* Lean mass in the left leg, *FMA-R* Fat mass in the right arm, *FMA-L* Fat mass in the left arm, *FMT* Fat mass in the trunk, *FML-R* Fat mass in the right leg, *FML-L* Fat mass in the left leg.

 FMT in women decreased significantly more in the treatment group than in the comparative group (− 5.85 [− 9.7; − 4.2] vs − 3.15 [− 4.9; − 2.5] kg, *p* = 0.001). FMT also decreased in males (− 7.8 [− 9.4; − 6.5] vs − 3.85 [− 1.65; − 0.8] kg, *p* < 0.001), as did FMA-R (− 1.1 [− 1.5; − 0.7] vs − 0.7 [− 1.0; − 0.4] kg, *p* = 0.024) and FMA-L (− 1.0 [− 1.4; − 0.7] vs − 0.7 [− 1.0; − 0.4] kg, *p* = 0.049).

**Figure 1:**
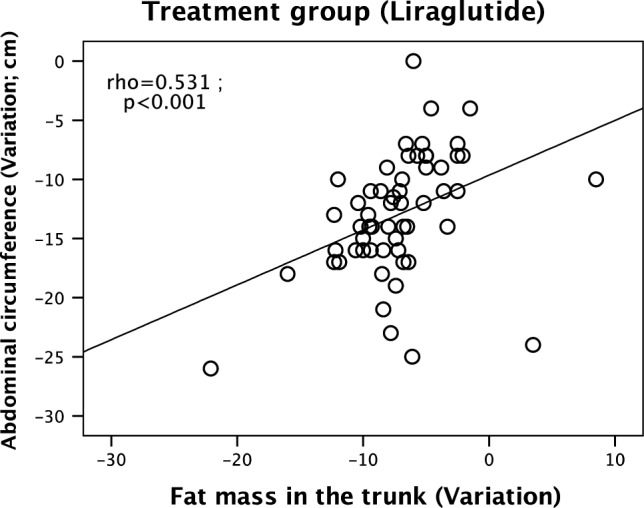
Correlation between reduction in abdominal circumference (AC) and reduction in fat mass in the trunk (FMT) after 6 months of treatment with liraglutide.

Since the reduction in AC and FMT was significantly greater in the treatment group than in the comparative group, we tested for a possible association between the two parameters and found a strong correlation (*rho* = 0.531; *p* < 0.001) in the treatment group (Fig. 1).

Finally, the multivariate approach revealed that treatment with liraglutide was independently associated with changes in FMT and BMI (Table 6).

**Table 6:** Multiple regression analysis to verify the independent association between liraglutide treatment with the reduction of fat mass of the trunk after 6 months.

	Reduction in fat mass in the trunk after 6 months			
	Initial model		Final model*	
	Beta non-standard coefficient (CI 95%)	*p*	Beta non-standard coefficient (CI 95%)	*p*
Liraglutide treatment	− 1.805 (− 3.567; − 0.043)	0.045	− 1.936 (− 3.462; − 0.411)	0.013
Sex, male	− 0.331 (− 2.395; 1.732)	0.751	–	–
Age, years	0.054 (− 0.016; 0.124)	0.127	–	–
SAP (mmHg)	− 0.088 (− 0.205; 0.029)	0.137	–	–
DAP (mmHg)	0.119 (− 0.07; 0.308)	0.215	–	–
BMI (kg/m^2^)	− 0.397 (− 0.707; − 0.087)	0.013	− 0.321 (− 0.473; − 0.17)	< 0.001
AC (cm)	0.046 (− 0.075; 0.168)	0.450	–	–
WHR (cm)	− 3.009 (− 17.824; 11.806)	0.688	–	–
HDL (mg/dL)	0.021 (− 0.021; 0.063)	0.320	–	–
Glycated hemoglobin (%)	− 0.258 (− 1.092; 0.576)	0.540	–	–
CRP (mg/dL)	0.165 (− 0.26; 0.591)	0.442	–	–

*SAP* Systolic arterial pressure, *DAP* Diastolic arterial pressure, *BMI* Body-mass index, *AC* Abdominal circumference, *WHR* Waist-to-hip ratio, *HDL* High density lipoprotein, *CRP* C-reactive protein.

*The method used to achieve the final model was the stepwise with backward approach.

## Discussion

Liraglutide at a daily dose of 3 mg was associated with weight loss and considerably improved clinical and laboratory findings in obese MetS pacients of both sexes, confirming the findings of previous trials^23,24^. BIA parameters (especially FMT) were significantly reduced in our sample of patients stratified by sex, and were correlated with AC. In addition, body weight, fat mass and FMA decreased significantly in males.

The use of an age- and sex-matched comparative group allowed us to reliably establish whether the use of a GLP-1 analog can significantly modify the BIA parameters of obese MetS patients of both sexes. In addition, the overall clinical, laboratory, anthropometric and BIA findings allowed to establish the effect of liraglutide on the cardiometabolic profile with 6 months of follow-up in obese patients with MetS, stratified by sex.

Six months of liraglutide treatment led to reductions in SBP and DBP and in all anthropometric variables in both sexes, matching several other studies^23–29^. In support of our findings, a double-blind study involving 3731 patients reported weight loss and a reduction of glycemia and cardiometabolic risk factors after 52 weeks of treatment with liraglutide at 3 mg/day^29^, suggesting the compound can significantly reduce insulin resistance and glycemia and promote weight loss.

Among the laboratory variables, improvement was observed for fasting glycemia, glycated hemoglobin, insulin resistance, TC, triglycerides and inflammatory markers, indicating a better overall metabolic and inflammatory profile^2,25,28,30^. Importantly, our findings point to a significantly improved cardiometabolic and inflammatory profile after 6 months of treatment, whereas other studies have generally relied on longer follow-up periods (~ 1 year)^28^, suggesting the possibility of an earlier onset of the effects of liraglutide, including weight loss and glycemia reduction.

Interestingly, we observed a reduction in anthropometric and BIA variables after 6 months of liraglutide treatment. Moreover, the relationship appeared to be sex-specific (men: BW, FM, AC, FMT, FMA-R and FMA-L; women: AC and FMT). In addition to the well-estabished abdominal adiposity in MetS patients of both sexes, in males BIA arm parameters also seem to reflect response to liraglutide in the cardiometabolic profile. Thus, the assessment of body composition by BIA may be influenced by sex and the subject’s level of hydration and obesity^13^.

Visceral adipose tissue is now known to be a key component of MetS. AC is therefore an important parameter in the clinical stratification of cardiometabolic risk. However, a high AC value alone is not enough to adequately assess the accumulation of abdominal fat^4,31^, making it necessary to adopt more accurate methods of quantification, capable of monitoring treatment and preventing cardiac complications.

BIA has been validated for the assessment of body composition^32–34^. The method can evaluate FM in several body compartments and has been shown to perform quite well compared to more costly methods, such as computed tomography^35^. In this study, BIA was used to assess different body segments, showing truncal fat loss to be correlated with reductions in AC and abdominal fat loss. Interestingly, a Brazilian study evaluated the reliability of BIA and indirect calorimetry in the measurement of the resting metabolic rate of 40 women with MetS over a period of 6 months and concluded that, compared to indirect calorimetry, BIA is a practical and time-saving method which does not require prolonged fasting in order to produce reliable results^36^.

The observed reduction in AC and FMT in patients treated with liraglutide implies a reduction in visceral fat—the main cardiovascular risk factor in MetS^4,31^. This is supported by the fact that liraglutide treatment remained independently associated with the BIA parameter FMT in the multiple regression analysis, suggesting BIA is an adequate tool of abdominal fat assessment.

The limitations of this study included the short follow-up period (6 months) and the relatively small sample of patients. Also, we did not submit patients to nutritional assessment, and at baseline almost all the anthropometric variables were higher in the treatment group than in the comparative group, suggesting a possible sampling bias. Thus, since it was not possible to reliably establish the difference between treatment and control, we repeated the analysis segregating the patients by sex and ran multiple regressions to confirm the independent association between liraglutide treatment (independent variable) and the reduction in FMT after 6 months (dependent variable).

In conclusion, treatment with liraglutide at 3 mg/day for 6 months promoted weight loss, improved cardiometabolic and inflammatory parameters and led to a significant reduction in FMT correlated with AC in obese MetS patients of both sexes. Studies on larger samples and with longer follow-up periods are necessary to confirm and extrapolate our findings.

## Data Availability

The datasets used and analyzed during the current study are available from the corresponding author on reasonable request. The data are not publicly available as they contain confidential information that may compromise the privacy/consent of the participants.
